# Magnetic Resonance Metabolic Profiling of Breast Cancer Tissue Obtained with Core Needle Biopsy for Predicting Pathologic Response to Neoadjuvant Chemotherapy

**DOI:** 10.1371/journal.pone.0083866

**Published:** 2013-12-19

**Authors:** Ji Soo Choi, Hyeon-Man Baek, Suhkmann Kim, Min Jung Kim, Ji Hyun Youk, Hee Jung Moon, Eun-Kyung Kim, Youn Ki Nam

**Affiliations:** 1 Department of Radiology, Research Institute of Radiological Science, Yonsei University College of Medicine, Seoul, Korea; 2 Department of Radiology, Samsung Medical Center, Seoul, Korea; 3 Division of Magnetic Resonance, Korea Basic Science Institute, Chungbuk, Korea; 4 Department of Bio-Analytical Science, University of Science and Technology, Daejeon, Korea; 5 Department of Chemistry and Chemistry Institute for Functional Materials, Pusan National University, Busan, Korea; 6 Agilent Technologies Korea, Seoul, Korea; National Research Council of Italy, Italy

## Abstract

The purpose of this study was to determine whether metabolic profiling of core needle biopsy (CNB) samples using high-resolution magic angle spinning (HR-MAS) magnetic resonance spectroscopy (MRS) could be used for predicting pathologic response to neoadjuvant chemotherapy (NAC) in patients with locally advanced breast cancer. After institutional review board approval and informed consent were obtained, CNB tissue samples were collected from 37 malignant lesions in 37 patients before NAC treatment. The metabolic profiling of CNB samples were performed by HR-MAS MRS. Metabolic profiles were compared according to pathologic response to NAC using the Mann-Whitney test. Multivariate analysis was performed with orthogonal projections to latent structure-discriminant analysis (OPLS-DA). Various metabolites including choline-containing compounds were identified and quantified by HR-MAS MRS in all 37 breast cancer tissue samples obtained by CNB. In univariate analysis, the metabolite concentrations and metabolic ratios of CNB samples obtained with HR-MAS MRS were not significantly different between different pathologic response groups. However, there was a trend of lower levels of phosphocholine/creatine ratio and choline-containing metabolite concentrations in the pathologic complete response group compared to the non-pathologic complete response group. In multivariate analysis, the OPLS-DA models built with HR-MAS MR metabolic profiles showed visible discrimination between the pathologic response groups. This study showed OPLS-DA multivariate analysis using metabolic profiles of pretreatment CNB samples assessed by HR- MAS MRS may be used to predict pathologic response before NAC, although we did not identify the metabolite showing statistical significance in univariate analysis. Therefore, our preliminary results raise the necessity of further study on HR-MAS MR metabolic profiling of CNB samples for a large number of cancers.

## Introduction

Neoadjuvant chemotherapy (NAC) is well established as a standard treatment for locally advanced breast cancer [1-3]. The use of NAC makes primarily inoperable tumors suitable for surgery, and allows more patients to undergo breast-conserving surgery instead of mastectomy [4]. The heterogeneous character of breast cancer, however, results in varied responses to NAC [5,6]. Pathologic complete response (pCR), which is obtained in less than 30% of patients receiving NAC [7], is strongly associated with improved long-term outcomes and has been suggested as a prognostic indicator [2,8,9]. In contrast, NAC could be ineffective in patients with significant residual disease by surgical pathology, considering the substantial toxicity of NAC regimens [10]. Further local and systemic therapy should be carefully considered for a subgroup of patients according to the residual burden of disease. Although pathologic response information could be used as a prognostic indicator or a guide for further treatment after surgery, it may not be available before surgical removal of the tumor. Pretreatment prediction of pathologic response to NAC could enable development of personalized treatment protocols, reducing unnecessary exposure of patients to chemotherapy toxicity and improving long-term patient outcome. 

Several studies have focused on identifying reliable markers to predict pCR in breast cancer patients receiving NAC. Some researchers have reported that the change in tumor size (diameter/volume) assessed by dynamic contrast enhanced magnetic resonance imaging (MRI) early during NAC treatment (i.e. size measurement after first or second cycles of treatment) could be a predictor of pCR [11,12]. Diffusion weighted imaging (DWI) and in vivo proton magnetic resonance spectroscopy (MRS) have also been proposed to predict pCR to NAC [12-14]. Other studies have shown that overexpression/amplification of HER2 (a receptor for human epidermal growth factor) and lower expression levels of estrogen receptor (ER) were associated with pCR [15-17]. However, to date, there is no powerful marker for predicting pCR before starting NAC treatment or early in NAC treatment. 

Ex vivo high-resolution magic angle spinning (HR-MAS) MRS provides highly resolved spectra of tissue samples. In addition, it requires less sample treatment and does not damage tissue integrity. The HR-MAS MR spectra of tissue samples consist of numerous peaks that reflect their metabolic composition. Metabolic profiling of numerous HR-MAS spectral data using multivariate statistical analysis can provide a way to analyze complex samples such as human tissues, and can be used for a non-targeted analysis to identify surrogate markers to predict the malignant transformation or treatment response. Recent studies have shown that HR-MAS metabolic profiling of tissue samples may be used for diagnoses or treatment monitoring of several human diseases, because HR-MAS MRS can display metabolic alteration of the tissue in response to external stress [18,19]. The assessment of metabolic composition by HR-MAS MRS has been applied in studies of breast cancer, and could be a promising approach for the diagnosis and characterization of breast cancer [20-24]. Recent studies have also reported that HR-MAS MR metabolic profiles could assist monitoring of treatment response to NAC and prediction of long-term survival in locally advanced breast cancer patients [25,26]. However, these studies conducted HR-MAS MRS using surgically obtained tissue specimens. Therefore, their results may not be directly applicable to the preoperative decision making stage concerning the best treatment approach for breast cancer patients. 

Percutaneous image-guided core needle biopsy (CNB) is a minimally invasive standard procedure for the diagnosis of breast cancer before surgery [27]. Breast cancer samples obtained by CNB are clinically important not only for pathologic diagnosis but also for immunohistochemical (IHC) analysis of histologic prognostic factors such as hormone receptor status [28]. Recent studies have shown that HR-MAS MRS using breast tissue samples obtained with CNB could differentiate cancer from non-cancer samples and predict tumor aggressiveness prior to surgery, by quantification of choline-containing compounds [29,30]. The purpose of our study was to determine whether metabolic profiling of CNB samples using HR-MAS MRS could be used for predicting pathologic response to NAC in patients with locally advanced breast cancer. 

## Materials and Methods

### Patients and sample preparation

 This study was approved by the institutional review board of Yonsei University College of Medicine, and written informed consent was obtained from all patients. 

Between October 2009 and November 2011, 109 patients with 114 breast lesions assessed by the Breast Imaging Reporting and Data System as stage 4c or 5 and larger than 1 cm in diameter on mammographic or ultrasound (US) were initially enrolled. We obtained the breast tissue sample for each lesion when these patients underwent US-guided CNB for pathologic diagnosis. The criteria for selection among these initial patients included: 20 years of age or older; having a breast lesion pathologically diagnosed as malignant by core biopsy; treated with NAC and underwent subsequent surgery; and not pregnant at the time of diagnosis. Finally, 37 patients with 37 locally advanced breast cancers (mean age 50.5 years; age range 30-67 years) fulfilled the inclusion criteria. 

 For each patient, one of four radiologists (with 6-13 years of experience) performed US-guided CNB using a 14-gauge dual-action semiautomatic core biopsy needle (Stericut with coaxial guide; TSK Laboratory, Tochigi, Japan). In patients with large and heterogeneous cancers, the homogeneously solid areas were targeted for biopsies. The mean number of tissue samples obtained by US-guided CNB was six (range 5-8) samples. All samples except for one core of each lesion were used for pathologic diagnosis and IHC analysis. For HR-MAS MRS, one CNB sample was placed in a cryogenic vial and immersed in liquid nitrogen immediately after biopsy. CNB samples were stored at -162°C for one to five months prior to HR-MAS MRS. All 37 patients were diagnosed with locally advanced cancer based on the CNB result and imaging findings obtained from breast US, mammography, and MRI. They were treated with anthracycline- and/or taxane-based NAC, and subsequently underwent surgery. 

### Histopathologic analysis

 All 37 breast lesions were pathologically diagnosed as malignant by CNB performed before NAC. After completion of NAC, the final pathologic diagnosis and the residual tumor size were established surgically in all patients. The pathologic response to NAC was assessed by comparing tumor size measured using breast imaging prior to NAC and that measured using the surgical specimen. In this study, pCR was defined as no invasive cancer present including two categories: no residual malignancy and no invasive cancer cell, but presence of ductal carcinoma in situ [31]. Axillary lymph node status was not taken into consideration for this analysis. When a residual tumor was present, the cases were classified into three categories: partial response (PR) with at least 30% size reduction, progressive disease (PD) with at least 20% size increase, and stable disease (SD) with neither sufficient shrinkage for PR nor sufficient increase for PD [32]. Histologic grade, ER, progesterone receptor (PR), HER2, Ki-67, and lymph node metastasis data were based on pathologic reports of CNBs performed before NAC. The histologic grade of each cancer lesion was determined with modified Bloom-Richardson classification [33]. ER and PR positivity was defined as more than 10 fmol/mg cytosol protein, or as 10% or more nuclear IHC staining. HER2 IHC using the HercepTest TM (DAKO) was interpreted as 0, 1+, 2+, or 3+, and was defined as positive in cases with 2+ or 3+ according to the ASCO/CAP guidelines [34]. IHC staining of Ki-67 was scored by counting the number of cells with positively stained nuclei and was expressed as a percentage of the total tumor cells. Staining results for Ki-67 were classified as follows: low, 0–29%; high, ≥ 30% [29].

### HR-MAS MRS experiments

 HR-MAS MRS was performed on the CNB specimens with an NMR (nuclear magnetic resonance) spectrometer (Agilent, VNMRS 500) operating at a proton NMR frequency of 500.13 MHz (11.74 T). The temperature was set to 19°C after calibration with methanol. Frozen samples were thawed in the NMR laboratory, weighed, and placed in an HR-MAS nano-probe® (Agilent, Walnut Creek, CA, USA). The total volume of the sample cell was 40 μl, and an average of 11.1 mg core-biopsy samples were placed in the cell with the remaining volume filled with D_2_O containing 0.01% trimethylsilyl propionic acid (TSP). An inverse-detection type probe equipped with a single Z gradient coil was used. The CNB tissue samples were analyzed using a CPMG (Carr-Purcell-Meiboom-Gill) pulse sequence to impose a T2 filter. All data were collected at a spinning rate of 2 kHz. The spectral acquisition parameters were as follows: 16K complex data points, 7961 Hz sweep width, 1.2 s acquisition time, 1.0 s relaxation delay, 1.5 s pre-saturation time (3.7 s total time of repetition (TR)), 1.0 ms inter-pulse delay (2 ms time of echo (TE)), 128 number of transients, 30 receiver gain and total acquisition time of 10 min. For the metabolite quantification, adequately long TR and short TE were used in order to neglect the T1/T2 relaxation time difference among metabolites and the TSP. 

**Figure 1:**
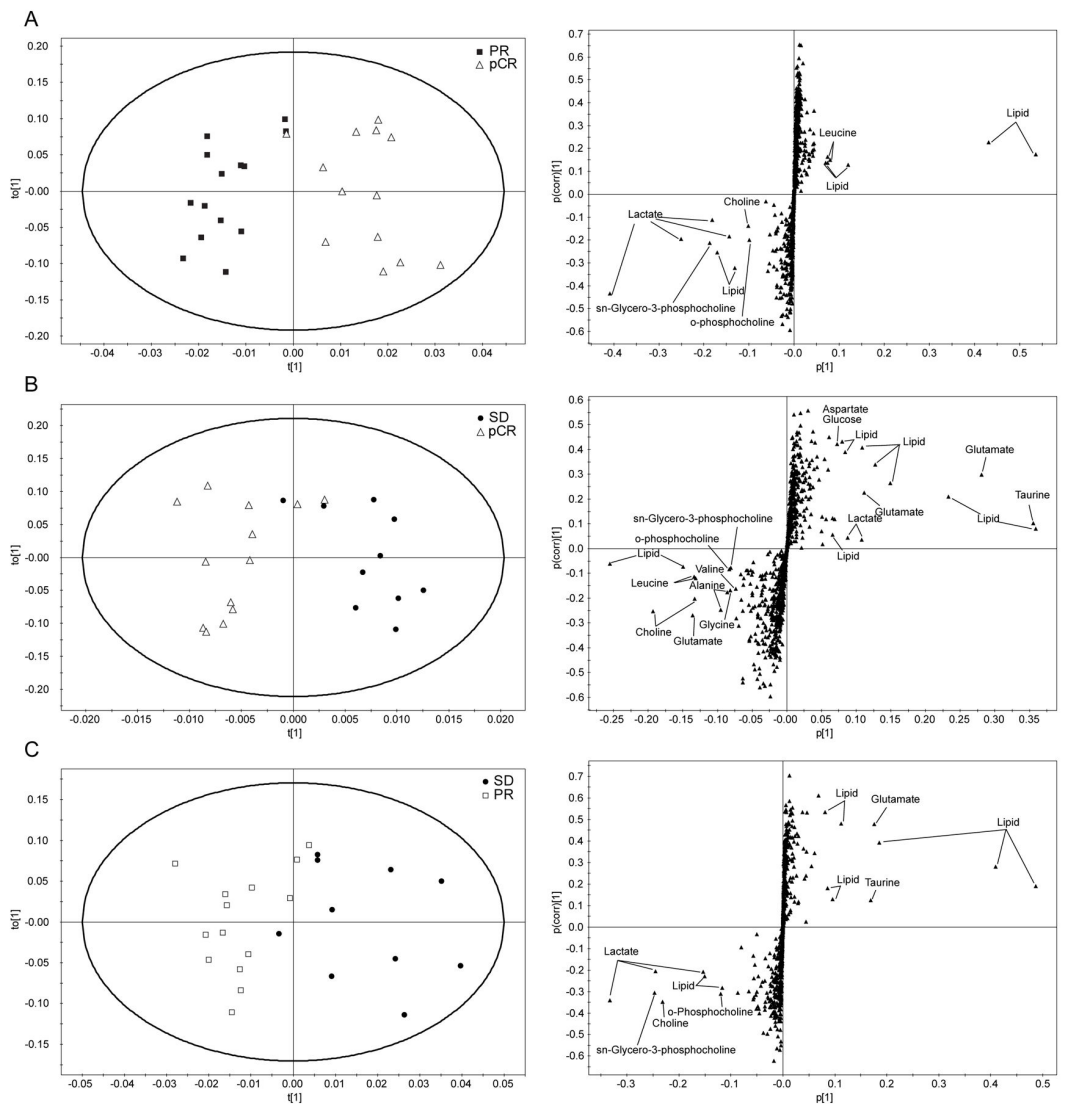
OPLS-DA score and loadinig S-plots of the HR-MAS MR spectra for predicting pathologic response to NAC. (A) pCR vs. PR (B) pCR vs. SD (C) PR vs. SD. pCR: pathologic complete response; PR: partial response; SD: stable disease.

The spectra were processed and analyzed using ACD software (Advanced Chemistry Development, Toronto, Ontario, Canada). Post-processing consisted of Fourier transformation, phasing and baseline correction. Chemical shifts were referenced in relation to the creatine (Cr) signal at 3.04 ppm. Spectral regions from 1.47 to 3.60 ppm [alanine (Ala), Cr, free choline (Cho), phosphocholine (PC), glycerophosphocholine (GPC), myo-inositol (m-Ins), taurine (Tau), and glycine (Gly)] were selected for quantification (Figure 1). The peak amplitudes of metabolites were measured by fitting a Voigt (e.g., Gauss+Lorentz) line-shape function. The integration values were normalized to the number of contributing protons per molecule and to tissue weight. Quantification was performed by comparing the integrated TSP signal to the signal of interest in the tumor spectrum. Absolute concentrations were recorded as µmol/g wet weight.

### Data and Statistical analysis

 Clinicopathologic characteristics of the included patients and tumors were collected from a review of patients’ medical records, and are listed in Table 1. Tumor size was measured with US or MRI performed before NAC treatment. 

**Table 1:** Clinicopathologic characteristics of the 37 patients with 37 locally advanced breast cancers in this study.

Patient characteristics		Pathologic response		
		pCR (n=13)	PR (n=14)	SD (n=10)
Age (mean ± S.D.)	years	51.2±9.7	48.1±8.8	52.9±9.7
Tumor size (mean ± S.D.)	mm	29.6±11.4	36.1±14.6	49.2±34.2
LN metastasis	positive	13	14	8
	negative	0	0	2
AJCC stage	II	11	10	5
	III	2	4	5
ER status	positive	9	9	7
	negative	4	5	3
PgR status	positive	1	3	2
	negative	12	11	8
HER2 status	positive	5	4	5
	negative	8	12	5
Ki-67 status	high	4	4	3
	low	9	10	7
Histologic grade	positive	2	4	3
	negative	9	10	7
	N/A	2	0	0

N/A: not available; S.D.: standard deviation.

pCR: pathologic complete response; PR: partial response; SD: stable disease.

 Spectral data acquired by HR-MAS MRS were expressed with metabolite concentrations [Ala, Cho, PC, GPC, total choline (tCho, the sum of Cho, PC, and GPC), Cr, Gly, Tau, m-Ins] and metabolic ratios (Cho/Cr, PC/Cr, GPC/Cr, GPC/PC, GPC/Cho, PC/Cho). Patients were grouped by pathologic response of the tumors to NAC. For classification of pathologic response to NAC, PR and SD groups were combined into a non-pCR group. The statistical differences of HR-MAS MR spectral data between the groups (pCR vs. PR vs. SD/pCR vs. non-pCR) were assessed using the Mann-Whitney test. Statistical analysis was performed with SAS for Windows, version 9.0 (SAS Institute, Cary, NC, USA). An adjusted *P* value of less than 0.0167 (Bonferroni corrected *P* = 0.05/3) was considered to indicate a significant difference between the three pathologic response groups. For comparison of pCR and non-pCR groups, a P value of less than 0.05 was considered to indicate statistical significance. 

For multivariate analysis of spectral data, Matlab (MathWorks, Natick, MA), SIMCA-P 11.0 (Umetrics, Sweden), and Excel (Microsoft, Seattle, WA) programs were used. Principal component analysis, partial least square discriminant analysis, and orthogonal projections to latent structure-discriminant analysis (OPLS-DA) were performed to distinguish patient groups by pathologic response to NAC with HR-MAS MR spectral data of CNB samples obtained before NAC. Class discrimination models were built until the cross-validated predictability value did not significantly increase to avoid over-fitting of the statistical model. The statistical model was validated by prediction of unknown samples using a leave-one-out analysis. An a priori cut-off value of 0.5 was used to evaluate the prediction results [35]. Signals contributing to group discrimination were identified by an S-plot and the corresponding HR-MAS MR spectral data were identified using Chenomx (Spectral database; Edmonton, Alberta, Canada) software and an in-house built database. The signals from the pollutants like ethanol and methanol were excluded from the statistical analysis of spectral data.

## Results

 Of the 37 invasive breast cancers in the 37 patients that were included in this study (mean age 50.5 years; range 30-67 years), the most common tumor type was ductal carcinoma (n=34), with other cancers being mucinous carcinoma (n=2) and papillary carcinoma (n=1). The mean tumor size was 37.4 mm (range 16-111 mm). Among the patients with residual disease at surgical pathology, no patients presented with PD. Therefore, the patients were divided into three groups (pCR, PR, and SD) according to their pathologic response to NAC, and 35.1% (13/37) of the included patients achieved pCR (Table 1). Although the clinicopathologic characteristics of each group were not statistically different, the pCR group showed a trend toward smaller size compared to the non-pCR (PR and SD) group.

 HR-MAS MRS was used to identify and quantify various metabolites in all 37 breast cancer tissue samples obtained by CNB (Table 2). The mean and medial values of tCho concentration were 1.18 µmol/g (range 0.003-5.626) and 0.67 µmol/g (interquartile range 0.069-1.978), respectively. In univariate analysis, the metabolite concentrations and metabolic ratios of CNB samples obtained with HR-MAS MRS were not significantly different between the pCR, PR, and SD groups (Table 3). In addition, HR-MAS MR spectral data were not significantly different between the pCR and non-pCR groups. However, there was a trend towards lower PC/Cr ratios in the pCR group compared to the non-pCR group, without statistical significance (*P*=0.077). 

**Table 2:** HR-MAS MRS values for 37 breast cancer specimens.

**Metabolite concentration (µmol/g)**			**Metabolic ratio**		
**Metabolite**	**Median (IQ range)**	**Mean (S.D.)**	**Ratio**	**Median**	**Mean**
Cho	0.15 (0.02-0.40)	0.31 (0.46)	Cho/Cr	2.55 (1.73-6.49)	4.84 (5.09)
PC	0.45 (0.06-1.12)	0.71 (0.82)	PC/Cr	1.79 (0.79-4.30)	6.32 (16.40)
GPC	0.06 (0.01-0.24)	0.16 (0.22)	GPC/Cr	1.15 (0.69-2.38)	1.97 (2.30)
tCho	0.66 (0.07-1.98)	1.18 (1.36)	tCho/Cr	7.48 (4.17-12.3)	13.14 (22.27)
Cr	0.22 (0.01-0.51)	0.32 (0.39)	GPC/PC	0.54 (0.35-1.03)	1.54 (3.91)
Gly	0.62 (0.03-0.76)	0.96 (1.17)	GPC/Cho	0.50 (0.19-0.92)	0.66 (0.65)
Tau	0.31 (0.03-0.78)	0.63 (0.90)	PC/Cho	0.78 (0.41-1.59)	1.30 (1.48)
m-Ins	0.05 (0.01-0.28)	0.29 (0.52)			
Ala	0.26 (0.03-0.48)	0.48 (0.62)			

Data represent the median (interquartile range, IQ) and the mean (standard deviation, S.D.).

Cho: choline; PC: phosphocholine; GPC: glycerophosphocholine; tCho: total choline (the sum of Cho, PC, and GPC); Cr: creatine; Tau: taurine; Gly: glycine; m-Ins: myo-inositol; Ala: alanine.

**Table 3:** Comparison of the HR-MAS MRS values according to pathologic response to NAC.

Metabolite or Metabolic ratio	Pathologic response							
	pCR (n=13)	PR (n=14)	SD (n=10)	Non-pCR (n=24)	pCR vs. PR	pCR vs. SD	PR vs. SD	pCR vs. non-pCR
	Median	Median	Median	Median	*P*	*P*	*P*	*P*
Cho	0.03 (0.006-0.524)	0.18 (0.054-0.518)	0.11 (0.004-0.541)	0.16 (0.033-0.373)	0.308	0.804	0.292	0.589
PC	0.12 (0.001-1.438)	0.66 (0.168-1.326)	0.21 (0.102-1.034)	0.57 (0.142-1.080)	0.145	0.577	0.320	0.215
GPC	0.04 (0.001-0.292)	0.09 (0.017-0.379)	0.06 (0.006-0.159)	0.07 (0.013-0.212)	0.174	0.951	0.198	0.356
tCho	0.19 (0.021-2.289)	0.99 (0.301-2.195)	0.74 (0.143-1.306)	0.97 (0.210-1.912)	0.207	1.000	0.219	0.408
Cr	0.05 (0.004-0.605)	0.23 (0.091-0.496)	0.20 (0.004-0.540)	0.23 (0.021-0.505)	0.528	0.951	0.725	0.656
Tau	0.05 (0.011-0.836)	0.58 (0.088-1.208)	0.28 (0.034-0.565)	0.40 (0.087-0.786)	0.167	0.733	0.208	0.279
m-Ins	0.02 (0.006-0.485)	0.17 (0.012-0.601)	0.05 (0.013-0.154)	0.12 (0.013-0.298)	0.308	0.804	0.253	0.426
Gly	0.03 (0.015-0.964)	0.48 (0.047-0.946)	0.27 (0.033-0.447)	0.34 (0.044-0.710)	0.396	0.804	0.266	0.494
Cho/Cr	5.13 (1.860-7.355)	3.32 (1.725-6.340)	2.01 (0.0730-6.143)	2.53 (1.380-5.943)	0.662	0.264	0.219	0.390
PC/Cr	1.41 (0.175-3.655)	2.59 (1.595-4.810)	2.00 (0.790-5.030)	2.38 (1.540-4.633)	0.047	0.368	0.380	0.077
GPC/Cr	1.15 (0.420-2.180)	1.89 (0.840-3.583)	0.89 (0.675-1.923)	1.26 (0.800-3.228)	0.109	0.877	0.197	0.332
tCho/Cr	7.63 (5.565-12.525)	7.83 (5.873-15.613)	4.15 (2.778-12.660)	6.80 (3.720-14.007)	0.627	0.313	0.241	0.824

Data represent the median (interquartile range) value (µmol/g). PR and SD groups were combined into the non-pCR group.

**Figure 2:**
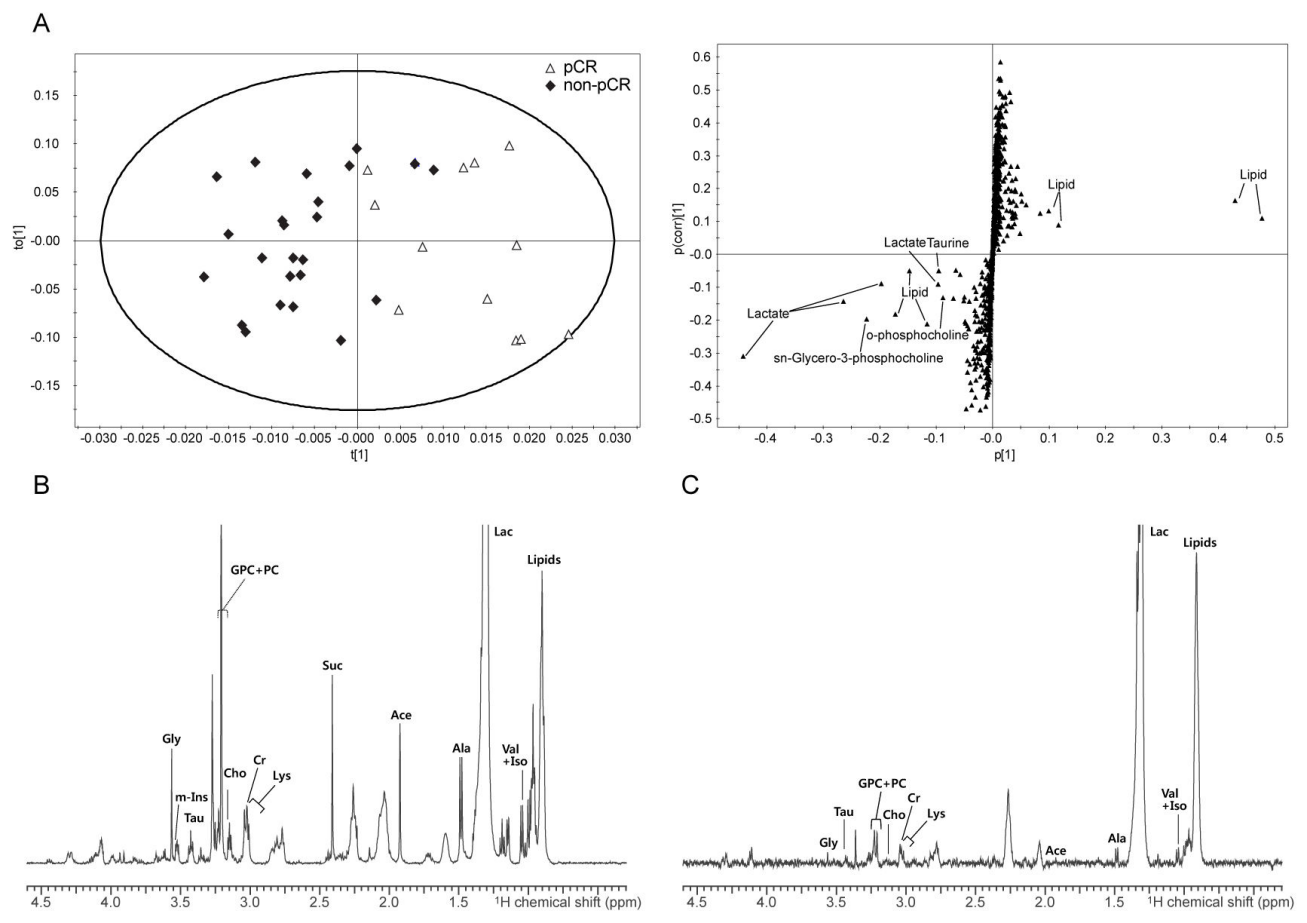
OPLS-DA score and loading S-plot of the HR-MAS MR spectra for differentiating pCR from non-pCR. (A) OPLS-DA score and loading S-plot of the HR-MAS MR spectra from pCR and non-pCR (PR and SD) groups. Representative spectra of the tumors showing pCR (B) and non-pCR (C) to NAC. pCR: pathologic complete response; PR: partial response; SD: stable disease. Cho: choline; PC: phosphocholine; GPC: glycerophosphocholine; tCho: total choline (the sum of Cho, PC, and GPC); Cr: creatine; Tau: taurine; Gly: glycine; m-Ins: myo-inositol; Ala: alanine; Suc: succinate; Lys: lysine; Ace: acetate; Val: valine; Iso: isoleucine.

For multivariate analysis, OPLS-DA separation models were built with the HR-MAS MR spectral data according to pathologic response to NAC. The OPLS-DA models showed visible discrimination between the groups by pathologic response to NAC, although some samples crossed over the reference line (Figure 1). In addition, an OPLS-DA score plot showed visible discrimination between pCR and non-pCR groups (Figure 2). Corresponding OPLS-DA loading S-plots showed that Tau, Cho, and GPC were contributing metabolites for the prediction of a pathologic response to NAC (Figure 1-2). Our OPLS-DA prediction model exhibited high sensitivities with range 84.6%–100% for differentiation pCR from other groups (Table 4). 

**Table 4:** Diagnostic performance of OPLS-DA for predicting pCR after neoadjuvamt chemotherapy.

	pCR vs. PR	pCR vs. SD	PR vs. SD*	pCR vs. non-pCR
Sensitivity	92.3 %	84.6 %	85.7 %	100 %
Specificity	100.0 %	90.0 %	90.0 %	87.5%

pCR: pathologic complete response; PR: partial response; SD: stable disease; non-pCR: PR and SD

* diagnostic performance for predicting PR

## Discussion

 In this study, we performed MR metabolic profiling of CNB tissue samples from patients with locally advanced breast cancer. HR-MAS MR spectra of our patient group were characterized with high concentrations of tCho, which is the sum of PC, GPC, and Cho. Choline-containing compounds are involved in biological functions such as cell signaling, lipid metabolism, and cell membrane integrity [36,37]. Many studies have reported that choline-containing compounds, especially PC, are elevated in breast cancer samples compared to non-cancer samples [21,23,24,38-40]. In addition, higher tCho concentrations have been detected in breast cancers with high tumor grade or higher pharmacokinetic parameters determined from dynamic contrast enhanced MRI [41,42]. The positive association between the concentration of choline-containing compounds and breast cancer tissue may be a consequence of up-regulation of choline kinase activity in response to demands from the cancer cell under hypoxic and angiogenic conditions, which are associated with aggressiveness of breast cancer [40,43,44]. In this study, there was a tendency of lower levels of the PC/Cr ratio and concentrations of choline-containing compounds in the pCR compared to the non-pCR group, without reaching statistical significance. The lower tendency of pretreatment tCho concentrations of the pCR group compared to the non-pCR group has also been observed in a previous study using in vivo proton MRS [45]. On the other hand, a recent study using HR-MAS MRS did not find a significant difference between pretreatment concentrations of choline-containing compounds in the PR and SD groups, although the metabolite concentrations of the pCR group was not evaluated due to a limited study population [26]. However, like our study, these previous studies did not show statistically significant differences in the concentrations of choline-containing compounds between different pathologic response groups. The lack of statistical significance may be due to the small numbers and different pathologic response features of the enrolled patient groups. Therefore, further studies with larger patient groups are needed to verify the association between choline-containing compounds and the pathologic response to NAC. 

Besides choline-containing compounds, previous studies reported that elevated concentrations of Tau and Gly can be associated with breast cancer tissue [23,24,26]. Tau is an amino acid associated with essential biological functions such as antioxidation, membrane stabilization, and apoptosis [12,46]. Gly is an amino acid involved in the control of protein synthesis, and an association between higher expression of the mitochondrial Gly biosynthesis pathway and higher mortality of breast cancer patients has been reported [34]. Recent studies using HR-MAS MRS have shown that Tau and Gly concentrations of breast cancer tissue were associated with survival after NAC treatment and with several prognostic factors including tumor size, PR, and HER2 status [26,29]. However, we did not find statistical differences of Tau and Gly concentrations of our CNB samples between the pCR and non-pCR groups, although they showed a trend of lower levels in pCR compared to non-pCR groups. 

Pretreatment differentiation of responders from non-responders to NAC is clinically important to decide whether NAC is a proper therapeutic option for patients with locally advanced breast cancers. However, pretreatment prediction of the pathologic response to NAC is challenging in breast cancer research. Recent studies found that pretreatment ADC values assessed by DWI may be an indicator to distinguish between responders and non-responders [12,13]. In spite of these initial results, clinical application of breast DWI can be difficult due to its high sensitivity to imaging artifacts and limited spatial resolution. In addition, DWI protocols including *b* values, which influence the ADC value, differ between institutions [12]. In these circumstances, our results using OPLS-DA multivariate analysis seem promising for prediction of patients’ pathologic response before NAC treatment. In cancer metabolomics, OPLS-DA has proven useful for classifying data with large intra-group variations such as the MRS data [29,30,46]. In our study, OPLS-DA models using HR-MAS MR spectral data of pretreatment CNB cancer samples provided visible discrimination between pCR and non-pCR groups. These results suggest that MR metabolic profiling of CNB cancer samples may be used as an indicator to predict pCR before NAC treatment. 

Although we did not find a statistical difference in clinicopathologic characteristics between the pathologic response groups, tumors of the pCR group tended to be smaller than those of the non-pCR group. Several clinical and pathologic factors have been shown to be associated with a better response to NAC. These include ER/PR negative status, high tumor grade, high proliferative activity, and smaller tumor size [47,48]. Among these, ER/PR negative status is considered a useful predictor for pCR in patients receiving NAC, because many studies have reported a significant correlation of ER/PR negative status with achieving pCR after NAC [49-51]. A recent study using HR-MAS MRS showed that tissue samples of human triple negative breast cancer had a higher GPC/PC ratio than samples of human ER/PR positive cancer [52]. This study also found higher GPC than PC concentrations in basal-like xenografts, whereas this pattern was reversed in luminal-like xenografts. These findings mean that different intrinsic subtype classified IHC analysis may have different choline metabolic profiles. Consequently, it is conceivable that metabolic profiles of choline-containing compounds may be not only a predictor of pathologic response to NAC but also a basis of a better understanding of differences in the metabolic mechanism between pCR and non-pCR groups. Therefore, further studies of both the differences in metabolic profiles according to intrinsic subtype of breast cancer, and the role of metabolic profiles in each subtype, will be helpful to identify predictors of pathologic response to NAC.

 Many previous studies using HR-MAS MRS have used surgically obtained tissue samples [23-26]. Therefore, the metabolic profiles could not be used to directly influence the pretreatment planning of therapeutic strategies. We conducted HR-MAS MRS using 14-gauge CNB samples and performed metabolic profiling of breast cancer without any problem. US-guided CNB is the most frequently used method for diagnosis of suspicious breast lesions and for IHC analysis for lesion characterization. Accordingly, metabolic profiles of CNB samples can be clinically applicable for pretreatment prediction of NAC response or prognosis. In addition, HR-MAS MRS does little damage to tissue integrity during examination, and therefore CNB samples can be re-used for later histopathologic examinations after HR-MAS MRS [20,30]. However, metabolic profiling using HR-MAS MRS requires an invasive procedure to obtain tissue samples (e.g., surgical excision, CNB, blood sampling). Therefore, some researchers have used in vivo proton MRS for acquiring metabolic information about breast cancers. In vivo proton MRS is a noninvasive method that can provide metabolic information about tumors, but a technique for adequate shimming and accurate voxel placement is necessary to acquire MR spectra of sufficient quality [53]. Also, this adjunctive method requires at least an additional 10 minutes to be added to the existing breast MRI examination time, which affects patient comfort and suitability. In consideration of these technical and clinical aspects, we believe that HR-MAS MRS using CNB samples is not inferior to in vivo MRS as an adjunctive method for metabolic profiling of breast cancer. A recent study evaluated the utility of in vivo proton MRS for predicting NAC response in breast cancer patients, and reported that the pretreatment tCho values obtained with in vivo proton MRS were not significantly different between the pCR and non-pCR groups [13]. On the other hand, our results, especially using OPLS-DA analysis, suggested that metabolic profiles of CNB samples using HR-MAS MRS may be used as a predictor of NAC response. Moreover, HR-MAS MRS using high magnetic field strength (11.7 T) could also be used to analyze individual choline-containing compounds, other metabolic markers such as Tau and Gly, and metabolic ratios, which showed significant associations with prognostic factors of breast cancer in previous studies [29,30]. In the recent studies using tissues from bladder cancer and head and neck squamous cell carcinoma, HR-MAS MRS using high magnetic field strength also showed multiple metabolic alterations, which include increased levels of choline-containing compounds and several amino acids compared to normal tissues [54-56]. Although we did not find statistical significance in this study, our HR-MAS MRS results also showed the differences in the levels of the aforementioned potential biomarkers according to its pathologic response to NAC. Considering previous studies with our own results, HR-MAS MRS using breast tissue acquired with minimally invasive CNB may be a clinically useful method to predict NAC response and to develop more personalized treatment protocols for locally advanced breast cancer patients, with respect to invasiveness and data quality. In addition, if in vivo proton MRS could be applied at a high magnetic field strength (7.0 T) with future technological improvements, the metabolic profiles of CNB samples using HR-MAS MRS could be the foundation for future research regarding in vivo high-field MRS. 

We note that our study had several limitations. First, we excluded small tumors with diameters less than 1 cm and included a relatively small number of patients, which may have affected the results. Therefore, further studies with large patient cohorts are necessary for validation of our multivariate classification models. Second, we did not assess the associations among metabolic profiles by HR-MAS MRS, pathologic response to NAC, and long-term outcomes such as survival. However, previous studies have already shown that tumor metabolic profiles by HR-MAS MRS could potentially assist in the prediction of long-term survival in locally advanced breast cancer patients [25,26]. Finally, we did not evaluate the association between pathologic response to NAC and intrinsic subtype by IHC analysis, which is considered as a predictor of NAC efficacy [49-51]. 

 In conclusion, this study showed that OPLS-DA multivariate analysis using choline-containing metabolites of pretreatment CNB samples assessed by HR-MAS MRS may be used to predict pathologic response before NAC treatment, although we did not identify the metabolite showing statistical significance in univariate analysis. Therefore, our preliminary results raise the necessity of further studies of HR-MAS MR metabolic profiling of CNB samples for a large number of cancers. In addition, we expect that HR-MAS MR metabolic profiling of pretreatment CNB samples may be helpful to develop more personalized treatment protocols for patients with locally advanced breast cancers.
